# Substance Use, Sleep Duration, and Health Among Adults in Ohio

**DOI:** 10.5888/pcd20.230198

**Published:** 2023-12-28

**Authors:** Estefania Hernandez, Stephanie Griggs

**Affiliations:** 1 Frances Payne Bolton School of Nursing, Case Western Reserve University, Cleveland, Ohio

## Abstract

**Introduction:**

Substance use affects approximately 46.3 million people aged 12 years or older (16.5% of the US population) and is associated with poor sleep health overall.

**Methods:**

We conducted a cross-sectional secondary analysis of data from the 2020 Behavioral Risk Factor Surveillance System survey in Ohio. The sample comprised 14,676 adults. We examined associations between the use of 2 types of substances (marijuana and nonprescribed prescription pain medication) and short sleep duration (<6 hours per night) and overall health (mental, physical, and general). We used linear and logistic regression modeling while adjusting for individual-level (age, sex, race and ethnicity, education, income, and body mass index) and area-level (socioeconomic deprivation) covariates.

**Results:**

Of survey respondents who answered questions, 9.2% (1,140 of 12,362) reported using marijuana, and 1.4% (111 of 8,203) used nonprescribed prescription pain medication. Respondents who used marijuana used it an average 17.3 days per month. In adjusted logistic regression models, the odds of reporting short sleep duration were 2.4 times greater among respondents who used nonprescribed prescription pain medication (vs those who did not). The odds of reporting short sleep duration, poor mental health, poor physical health, and poor general health were 1.5, 1.3, 2.1, and 1.9 times greater, respectively, among respondents who reported marijuana use (vs those who did not). In the linear regression models (adjusted), more days of marijuana use were associated with longer sleep duration, worse mental health, and worse general health.

**Conclusion:**

Understanding the connection between substance use and health outcomes is needed to improve trajectories of substance use and recovery. Sleep duration is often underassessed among people who use substances. Expanding diagnostics and treatment options for those who use substances may result in lower levels of substance use and improved overall health.

**Summary**

**What is already known on this topic?**

The relationship between substance use and disturbed sleep is complex and bidirectional. People who misuse substances often report unhealthy sleep-related behaviors.

**What is added by this report?**

We characterized the associations between short sleep duration and the use of 2 types of substances (marijuana and nonprescribed prescription pain medication) among adults living in Ohio.

**What are the implications for public health practice?**

Short sleep duration is often underassessed and underdiagnosed. A need exists to increase global interest and awareness in understanding how sleep duration can affect substance use behaviors and recovery and vice versa.

## Introduction

Substance misuse affects approximately 46.3 million people aged 12 years or older in the US (16.5% of the population) and is associated with poor sleep health overall (satisfaction, alertness, timing, efficiency, and duration) (1,2). More than one-third of adults do not achieve the 7 to 9 hours of sleep recommended by the National Sleep Foundation and report high rates of irritability, headaches, slow reaction time, hormonal changes, daytime sleepiness (hypersomnia), and poor overall health (3–5). To increase overall sleep duration and quality and next day alertness and sustain next-day medical, financial, and social responsibilities (eg, employment, friendships, family), people may self-medicate with alcohol, cannabis, and prescription and nonprescription pain medications (6,7).

The most common misused substances are cannabis (marijuana) (17.5% or 48.2 million people), followed by prescription pain relievers (3.5% or 9.7 million people), and prescription benzodiazepines (1.8% or 4.8 million people) among people aged 12 or older in the US (8). Among those who reported current alcohol use, 47% (65.8 million people) reported past-month binge drinking (8). Although substance use has increased over the years, the percentage of people diagnosed with a substance use disorder (ie, alcohol, cannabis, and prescription pain relievers) has remained stable from 2015 through 2019 (8).

Short sleep duration and substance use have a negative effect on emotional and mood regulation; therefore, a person’s mental health status is at risk (9–11). Mental health symptoms or conditions have a reciprocal relationship with sleep duration. For example, worse mental health symptoms are associated with shorter sleep duration and drug misuse, while sleep problems and drug use can further exacerbate mental health symptoms and affect recovery outcomes (12). However, the adverse consequences of short sleep duration due to substance use are not routinely assessed in clinical practice. Detoxification programs and mental health interventions should incorporate an assessment of sleep duration as a core function in primary care models.

The objective of this study was to quantify substance use (use of marijuana and nonprescribed prescription pain medication) and examine the associations among substance use, sleep duration, and health (mental, physical, and general) among a sample of adults residing in Ohio, while adjusting for individual (age, sex, race and ethnicity, education, income, and body mass index [BMI]) and area-level (socioeconomic deprivation) covariates. We used a conceptual model of sleep health to guide this study (13). We hypothesized that higher levels of marijuana and nonprescribed prescription pain medication use would be associated with short sleep duration, and poor physical, mental, and general health. 

## Methods

We conducted a cross-sectional secondary analysis of data from the 2020 Behavioral Risk Factor Surveillance System (BRFSS). BRFSS is a system of health-related telephone surveys that collect data from civilian, noninstitutionalized US residents on their health-related risk behaviors, chronic health conditions, and use of preventive services (14). BRFSS collects data in all 50 states as well as the District of Columbia and 3 US territories. In addition, states have the option to add health questions as they pertain to the state; these questions help inform local programs and advocacy groups on the current scope of their interested issue or need. The state-added questions asked about prescription pain medication that had not been prescribed. The questions about marijuana use were asked as part of the core module. 

The Ohio BRFSS is conducted by the Ohio Department of Health with support from the Centers for Disease Control and Prevention (CDC). Data are available to state and local decision makers, legislators, researchers, students, and the general public. Data are collected from a random probability sample representative of the 14 geographic regions in Ohio; our analytic sample consisted of 14,676 adults. The institutional review board at Case Western Reserve University reviewed and approved the study (IRB no. 20221217).

### Variables and measures

We selected all covariates a priori on the basis of the scientific literature: age (years), sex at birth (male or female), race and ethnicity (Hispanic, non-Hispanic American Indian or Alaskan Native, non-Hispanic Asian, non-Hispanic Black, Non-Hispanic White, and non-Hispanic Other race), education (less than high school diploma, high school diploma, some college or technical school, college or technical school graduate), BMI, and social deprivation. The 6 domains of the composite Social Deprivation Index (SDI) are 1) income (percentage of the population living in poverty), 2) education (percentage with <12 years of education), 3) housing (percentage living in rented housing unit and percentage living in overcrowded housing unit), 4) household characteristics (percentage of single-parent households with dependents aged <18 y), 5) transportation (percentage of households without a car), and 6) employment (percentage of unemployed adults aged <65) (15). SDI data help shed light on various social determinants of health and are used to visualize and quantify socioeconomic differences in health outcomes by location. We used zip code tabulation areas (ZCTAs) based on generalized US Postal Service zip codes to calculate the SDI (15). The SDI is based on data from 5-year estimates from 2015–2019 (15). The BRFSS also asks about annual household income from all sources; we categorized these data into 7 categories of income, ranging from less than $15,000 to more than $75,000.

The independent variables of interest were 2 commonly used substances: marijuana and prescription pain medication not prescribed. The dependent variables of interest were self-reported sleep duration and health (physical, mental, and general).

**Sleep duration.** Self-reported sleep duration was a single-item BRFSS measure. Respondents were asked, “On average, how many hours of sleep do you get in a 24-hour period?” Responses ranged from 1 to 24 hours.

**Physical health.** Data on physical health were derived from the number of days the respondent reported that physical health was not good in the previous 30 days. Respondents selected 1 of the following response options: 0 days, 1 to 13 days, more than 14 to 30 days, or don’t know.

**Mental health.** Mental health status was self-rated in the BRFSS on the same 3 levels as physical health: 0 days, 1 to 13 days, or 14 to 30 days when mental health was not good. Other factors related to difficulty concentrating because of physical, mental, or emotional conditions were included by asking, “Do you have serious difficulty concentrating, remembering, or making decisions?” Respondents answered yes or no. Data on poor physical or mental health were derived by asking how many days in the past 30 days poor physical or mental health prevented the respondent from doing their usual activities, such as self-care, work, or recreation.

**General health.** Data on general health were derived from the BRFSS question that asked, “Would you say that in general your health is excellent, very good, good, fair, poor, or don’t know?” Respondents who reported having excellent, very good, or good health were allocated to the good or better health category. Respondents who reported having fair or poor health were allocated to the fair or poor health category.

**Substance use.** Frequency of marijuana use, method of use and purchase, and reason for use were addressed with questions such as, “During the past 30 days, on how many days did you use marijuana or hashish?” “During the past 30 days, which one of the following ways did you use marijuana the most often? Did you usually smoke it, eat it, drink it, vaporize it, dab it, use it some other way, or don’t know? When you used marijuana or cannabis during the past 30 days, was it usually for medical reasons, nonmedical reasons, for both medical and nonmedical reasons, or don’t know/not sure.” The survey did not ask about the use of electronic vaping products for marijuana use. Prescription pain medication was assessed with the question, “In the past year did you use prescription pain medication that was not prescribed to you?” Response options were yes or no.

### Statistical analysis

We used a quantitative descriptive approach to characterize substance use (prescription pain medication and marijuana use), sleep duration, and health (mental, physical, and general) among the study population. We used bivariate correlations, multivariable logistic regression models, and multivariable linear regression models to examine the relationships among substance use, sleep duration, and mental, physical, and general health.

To assess exposure in the logistic regression models, we dichotomized substance use into 2 groups (yes and no) for each substance (prescription pain medication and marijuana). We defined marijuana use as yes (1–30 days) or no (0 days). We defined short sleep duration as 6 hours or less (based on the National Sleep Foundation recommendations) and poor mental and physical health as 14 days or more of poor mental and physical health in the past 30 days (16–18). For the logistic regression models, we used the single item about prescription pain medication use (yes or no). For poor general health, we collapsed fair and poor into poor and compared this category with excellent, good, and very good. We calculated unadjusted and adjusted odds ratios (ORs) and adjusted for the following covariates: age, sex, race and ethnicity, education, income, BMI, and socioeconomic deprivation.

For linear regression, we used the single item about marijuana use: “During the past 30 days, on how many days did you use marijuana or hashish?” To evaluate explanatory contributions of substance use (marijuana) to sleep duration and health (mental, physical, and general), we performed a series of multivariable linear regression models. The linear regression models were run unadjusted (model 1) and adjusted with covariates (model 2): age, sex, race and ethnicity, education, income, BMI, and socioeconomic deprivation. Significance was set at *P* < .05.

## Results

The study population of 14,676 adults aged 18 to 99 (mean [SD], 53.6 [19.3]) years resided across 1,101 ZCTAs (Table 1). About half (54.3%) identified as female at birth, and most (85.0%) identified as non-Hispanic White, followed by 6.7% non-Hispanic Black, 2.2% non-Hispanic Asian, 2.1% other race, 1.9% non-Hispanic multiracial, and 2.1% Hispanic. The mean (SD) BMI was 28.9 (7.2). The mean (SD) number of poor mental health and physical health days were 4.5 (8.7) days and 12.4 (11.3) days, respectively.

**Table 1:** Characteristics of Study Population (N = 14,676), Ohio Behavioral Risk Factor Surveillance System Survey, 2020

Characteristic	No. of respondents who answered question	Value
**Age group, y, no. (%)**		
18–24	14,274	875 (6.1)
25–34		1,540 (10.8)
35–44		1,818 (12.7)
45–54		2,219 (15.5)
55-64		2,952 (20.7)
≥65		4,870 (34.1)
**Age, mean (SD), y**	14,274	53.6 (19.3)
**Sex, no. (%)**		
Female	14,676	7,970 (54.3)
Male		6,706 (45.7)
**Race and ethnicity, no. (%)**		
Hispanic	14,676	310 (2.1)
Non-Hispanic Asian		329 (2.2)
Non-Hispanic Black		983 (6.7)
Non-Hispanic White		12,468 (85.0)
Non-Hispanic multiracial		281 (1.9)
Non-Hispanic Other race		305 (2.1)
**Education**		
Less than high school diploma	14,626	903 (6.2)
High school diploma		4,932 (33.7)
Some college or technical school		4,068 (27.8)
College graduate		4,723 (32.3)
**Annual household income, no. (%), $**		
<15,000	14,382	1,051 (7.3)
15,000–19,999		939 (6.5)
20,000–24,999		1,172 (8.1)
25,000–34,999		1,261 (8.8)
35,000-49,999		1,704 (11.8)
50,000–74,999		1,897 (13.2)
≥75,000		3,666 (25.5)
**Social Deprivation Index, mean (SD)^a^**	13,527	47.0 (25.8)
**Body mass index, mean (SD), kg/m^2^**	13,314	28.9 (7.2)
**Sleep duration, mean (SD), hours**	14,449	7.0 (1.5)
**Mean no. (SD) of days of poor health in previous 30 days**		
Poor mental health	14,182	4.5 (8.7)
Poor physical health		12.4 (11.3)
Poor general health		12.6 (10.6)
**Mean no. (SD) of days of marijuana use of respondents who reported using marijuana**	12,342	17.3 (12.2)
**Substance use**		
Used marijuana	12,362	1,140 (9.2)
Used nonprescribed prescription pain medication	8,203	111 (1.4)
**General health**		
Excellent	14,644	2,686 (18.3)
Very good		4,925 (33.6)
Good		4,486 (30.6)
Fair		1,917 (13.1)
Poor		630 (4.3)

a The 6 domains of the composite Social Deprivation Index are 1) income (percentage of the population living in poverty), 2) education (percentage with <12 years of education), 3) housing (percentage living in rented housing unit and percentage living in overcrowded housing unit), 4) household characteristics (percentage of single-parent households with dependents aged <18 y), 5) transportation (percentage of households without a car), and 6) employment (percentage of unemployed adults aged <65) (15). The index is scaled from 1 to 100, with higher scores indicating higher levels of social deprivation.

Of the respondents who answered the questions, 1,140 (9.2%) reported using marijuana, and 111 (1.4%) used prescription pain medication not prescribed. People who reported use of nonprescribed prescription pain medication had 2.8 times and 2.4 times higher odds of short sleep duration (OR = 2.78; 95% CI, 1.80–4.30; adjusted OR [AOR] = 2.37; 95% CI, 1.50–3.76) in the unadjusted and adjusted models, respectively (Table 2), than people who did not report use of nonprescribed prescription pain medication. People who reported use of nonprescribed prescription pain medication had 1.7 times higher odds of poor general health in the unadjusted model than who did not report such use; however, the association was no longer significant after adjusting for covariates (age, sex, race and ethnicity, education, income, BMI, and socioeconomic deprivation). The associations between prescription pain medication use and mental or physical health were not significant.

**Table 2:** Results of Logistic Regression Models for Use of Nonprescribed Prescription Pain Medications, Marijuana, and Outcomes, Ohio Behavioral Risk Factor Surveillance System Survey, 2020

Outcome	OR (95% CI)	*P* value	Adjusted OR (95% CI)^a^	*P* value
**Use of nonprescribed prescription pain medication**				
Short sleep duration (<6 hours per night)	2.78 (1.80–4.30)	<.001	2.37 (1.50–3.76)	<.001
Poor mental health (>14 poor mental health days in past 30 days)	1.38 (0.91–2.11)	.13	1.37 (0.88–2.12)	.17
Poor physical health (>14 poor physical health days in past 30 days)	1.30 (0.75–2.25)	.35	1.06 (0.57–1.98)	.86
Poor general health (fair/poor health)	1.75 (1.14–2.66)	.01	1.60 (0.99–2.58)	.05
**Use of marijuana**				
Short sleep duration (<6 hours per night)	1.70 (1.46–1.97)	<.001	1.46 (1.24–1.73)	<.001
Poor mental health (>14 poor mental health days in past 30 days)	1.55 (1.36–1.78)	<.001	1.34 (1.16–1.55)	<.001
Poor physical health (>14 poor physical health days in past 30 days)	1.68 (1.43–1.98)	<.001	2.14 (1.78–2.57)	<.001
Poor general health (fair/poor health)	1.53 (1.33–1.75)	<.001	1.92 (1.64–2.24)	<.001

Abbreviation: OR, odds ratio.

a Covariates adjusted for age, sex at birth, race and ethnicity, education, annual household income, body mass index (individual level), and socioeconomic deprivation (area level).

People reporting use of marijuana had 1.7 times and 1.5 times higher odds of short sleep duration (OR = 1.70; 95% CI, 1.46–1.97; AOR = 1.46; 95% CI, 1.24–1.73), 1.6 and 1.3 times higher odds of poor mental health, 1.7 and 2.1 times higher odds of poor physical health, and 1.5 and 1.9 times higher odds of poor general health in the unadjusted and adjusted models than people not reporting use of marijuana, respectively (Table 2).

In the linear regression models, higher rates of marijuana use were associated with longer sleep duration (unadjusted, β = 0.93, *P* = .001, R^2^ = .009; adjusted, β = 0.08, *P* = .003, R^2^ = .016) (Table 3), worse mental health symptoms (adjusted, β =.077 , *P* = .01, R^2^ =.025), and poorer general health (adjusted, β = 0.06, *P* = .03 , R^2^ = 0.126) only after adjusting for covariates.

**Table 3:** Results of Linear Regression Models for Marijuana Use and Covariates to Sleep, Mental, and Physical Health Outcomes, Ohio Behavioral Risk Factor Surveillance System Survey, 2020

Model	Dependent variable	B (SE)	β	*P* value	R^2^
Model 1 (unadjusted)	Sleep duration	0.007 (0.002)	0.093	.001	0.009
	Mental health	0.027 (0.02)	0.055	.06	0.003
	Physical health	0.007 (0.01)	0.016	.58	0.000
	General health	0.002 (0.001)	0.031	.28	0.001
Model 2 (adjusted for covariates)^a^	Sleep duration	0.008 (0.003)	0.089	.003	0.016
	Mental health	0.04 (0.02)	0.077	.01	0.025
	Physical health	0.01 (0.01)	0.023	.45	0.050
	General health	0.003 (0.002)	0.063	.03	0.126

Abbreviations: B, unstandardized regression coefficient; β, standardized regression coefficient; R^2^, coefficient of determination.

a Covariates adjusted for age, sex at birth, race and ethnicity, education, annual household income, body mass index (individual level), and socioeconomic deprivation (area level).

## Discussion

We investigated the associations among substance use, sleep duration, and health (mental, physical, and general) among a sample of adults residing in Ohio while adjusting for individual and area-level covariates. People reporting use of nonprescribed prescription pain medication or marijuana had 3-fold and 2-fold higher odds of short sleep, respectively, even after considering both individual and area-level covariates. People reporting marijuana use had higher odds of poor health across all outcomes (sleep, mental, physical, and general health) even after considering covariates. These findings support the body of evidence that marijuana use is associated with mental health symptoms (19), irritability, sleep difficulties, anxiety, and learning and memory functioning deficits, particularly if use begins during adolescence or young adulthood (20). Moreover, substance use has been shown to exacerbate preexisting conditions (21).

Our findings align with previous evidence of the association between higher levels of substance use and shorter sleep duration and poorer general health overall (22). However, these associations vary and are often confounded by types of substances as well as underlying experiences and illnesses or treatment trajectories (23, 24). Our results indicate that people who use pain medication have higher odds of short sleep duration and higher odds of poor general health. The association between use of pain medication and odds of short sleep duration remained significant after accounting for the covariates age, sex, education, BMI, and socioeconomic deprivation. However, the association between use of pain medication and poor general health was no longer significant after adjusting for age, sex, education, BMI, and socioeconomic deprivation. These findings differ from other work that controlled for covariates and found that men were more likely than women to opt for alcohol; younger people were more likely than older people to opt for marijuana than alcohol; and people with low levels of education consumed more substances than people with higher levels of education (25,26).

Our findings also support the literature on the association between higher levels of marijuana use and poorer mental and general health (27). In our study, people who reported any marijuana use had higher odds of short sleep duration and more days of poor mental, physical, and general health in both the unadjusted and adjusted models. Our findings on marijuana use and short sleep duration are consistent with findings from a study based on data from the 2005–2018 National Health and Nutrition Examination Surveys in which people who reported using marijuana were 1.3 times more likely than people not reporting marijuana use to report short sleep duration (28). Our results are also consistent with the results of studies that showed an association between sleep disturbance and marijuana use among adolescents and adults (29,30). Contrary to our hypothesis, in our linear models, higher levels of marijuana use were associated with longer sleep duration. These findings are supported by findings of the NHANES study in which people who reported using marijuana were 1.6 times more likely than people not reporting marijuana use to report long sleep duration (28).

The 2020 Franklin County Community Needs Assessment in Ohio argued for prioritizing mental health and substance use programs because of the rising rate of emergency department admissions and the increasing demand for addiction specialists (31). The priorities did not include short sleep duration or sleep hygiene interventions. Sleep duration is a modifiable health behavior: improvement in this behavior could lead to decreases in rates of substance use and improve the health trajectories for people using substances. Sleep duration should be addressed in primary care and at the community level by expanding diagnostic and treatment options that cover a range of sleep problems and sleep disorders (eg, insomnia, sleep apnea, snoring, circadian rhythms, restless leg syndrome, narcolepsy). Services should help assess patients regardless of their age, sex and gender, race and ethnicity, or socioeconomic position. Community interventions based on an assessment of self-reported health, mental health, work–life balance, and sleep–wake habits may help identify people at high risk of inadequate sleep and how it may perpetuate other physical or behavioral health concerns.

The rate of unintentional deaths caused by drug use, including use of nonprescribed prescription pain medication, increased from 16.0 per 100,000 people in 2016 to 24.1 per 100,000 people in 2019 in Franklin County, Ohio (31). This rate is equal to approximately 24 residents dying annually due to nonprescribed drug or medication use — 36.8 times lower than the rate in Ohio overall but 19.7 times higher than the national average (8). New data provided by CDC’s National Center for Health Statistics indicate a substantial increase in overdose deaths from opioids, particularly synthetic opioids (fentanyl), psychostimulants such as methamphetamine, and semisynthetic opioids (nonprescribed prescription pain medication). Poverty, isolation, and social deprivation caused by COVID-19 and worsened socioeconomic hardship may have exacerbated existing drug and alcohol use. Socioeconomic hardship may also have led to increased periods of mental health symptoms and insufficient sleep, which may also have exacerbated existing substance use behaviors and encouraged unhealthy coping mechanisms. Therefore, addressing sleep disturbance and quality in the context of substance use may not only deter the increased prevalence of substance use but may also reduce the severity of substance use and poor health outcomes.

### Limitations

Our study has several limitations. First, we used self-reported data on sleep duration and substance use. Both are subject to social desirability bias, and both, according to previous research, may have been underestimated (32,33). However, data on self-reported sleep and substance use have been shown to be reliable (34). Our findings should be corroborated with objective measures of sleep duration and substance use. Second, the cross-sectional design of our study prevented us from investigating whether short sleep duration precedes substance use or vice versa. Third, survey respondents may have underestimated or overestimated mental, physical, or general health status. Finally, the racial and ethnic composition of our study population, which was primarily non-Hispanic White, may raise concerns about generalizability.

### Conclusions

Our research suggests a need to increase global interest and awareness in understanding how sleep duration can affect substance use behaviors and recovery and vice versa. Sleep duration is often underassessed and ultimately underdiagnosed among people who misuse substances. Furthermore, people with inadequate sleep duration may rely solely on willpower to mitigate poor or insufficient sleep without realizing it may be linked with another underlying chronic condition. If sleep problems or disorders are left untreated, people may experience difficulties in several areas of health such as memory, concentration, mood regulation, sex drive, and cardiovascular and immune system health.
